# COVID-19 related leukoencephalopathy with bilateral reticular formation involvement

**DOI:** 10.1259/bjrcr.20210054

**Published:** 2021-06-16

**Authors:** Islam Ahmed Hassan Ahmed, loai Aker, Mamdouh Sharafeldin, Ahmed Own, Mohamed Abdelhady, Surjith Vattoth

**Affiliations:** 1 Neuroradiology Fellow, Neuroscience Institute, Hamad Medical Corporation, Doha, Qatar; 2 Diagnostic Radiology Resident, Department of Clinical Imaging, Hamad Medical Corporation, Doha, Qatar; 3 Neuroradiology Consultant, Neuroscience Institute, Hamad Medical Corporation, Doha, Qatar; 4 Associate consultant neuroradiology, Neuroscience Institute, Hamad Medical Corporation, Doha, Qatar; 5 Associate Professor of Radiology, Neuroradiology Section, University of Arkansas for Medical Sciences (UAMS), Little Rock, AR, USA

## Abstract

We are presenting the imaging findings of COVID-19-related leukoencephalopathy associated with bilateral reticular formation diffusion restriction in brain magnetic resonance imaging. To the best of our knowledge, this is the first reported case of bilateral reticular formation affection in a COVID-19 patient.

## Case summary

A 44-year-old male, with history of diabetes mellitus and hypertension, presented to the emergency department with a progressive course of shortness of breath, cough and fever for 5 days. On examination, the patient was fully conscious and oriented, with a Glasgow Coma Scale of 15. He was tachypneic with a respiratory rate of 40–45 breaths/min and oxygen saturation that only improved from 54 to 80% with non-re-breather mask. The decision was made to intubate the patient considering his hypoxaemia and tachypnea. The patient then tested positive for COVID-19 polymerase chain reaction (PCR) from oropharyngeal and nasopharyngeal swabs.

The chest radiograph displayed bilateral pulmonary airspace opacities with no evidence of pleural effusion. Initial laboratory studies revealed elevated c-reactive protein, fibrinogen, lactate, liver enzymes and glucose level. However, complete blood count was unremarkable.

The patient received multiple medications including azithromycin, enoxaparin, esomeprazole, hydroxychloroquine, lopinavir/ritonavir, methylprednisolone, oseltamivir, piperacillin-tazobactam and paracetamol. He was maintaining oxygen saturation of 98% on mechanical ventilation.

**Figure 1.**
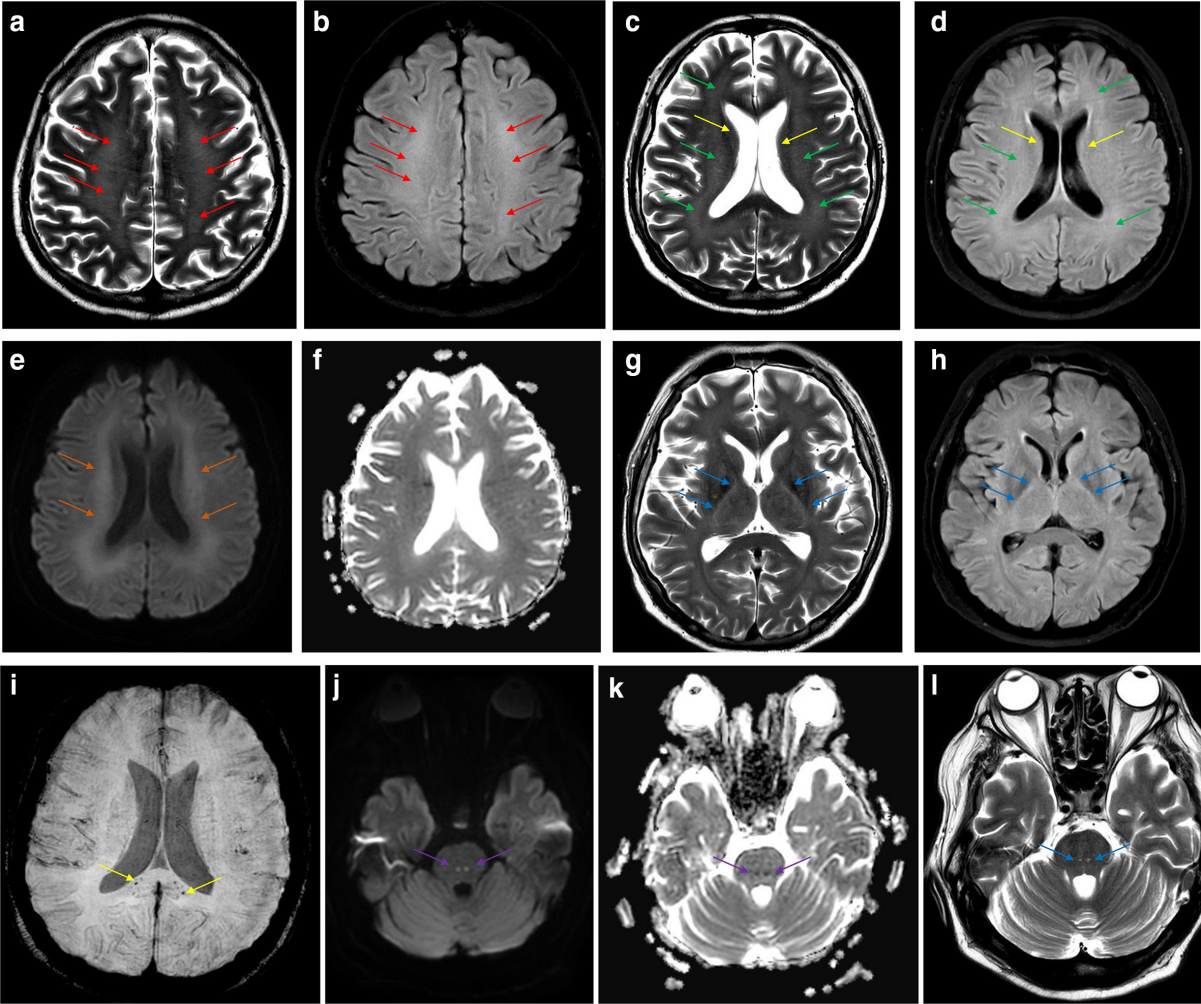
Axial T2W image (a) and FLAIR image (b) at the level of centrum semiovale showing bilateral symmetric centrum semiovale faint high signal intensity (red arrows). Axial T2W image (c) and FLAIR image (d) at the level of body of lateral ventricles show bilateral symmetric periventricular (yellow arrows) and corona radiata (green arrows) faint high signal intensity. Axial diffusion-weighted (e) and ADC images (f) at the level of lateral ventricle bodies showing corresponding regions of diffusion restriction (high DWI signal and low ADC value) at corona radiata bilaterally (orange arrows). Axial T2W image (g) and FLAIR image (h) at the level of basal ganglia show bilateral symmetric high signal intensity at posterior limb of both internal capsules (blue arrows). Susceptibility-weighted images (SWI) image (i) at the level of body of lateral ventricles showing minute dark blooming foci at splenium of corpus callosum (yellow arrows). Axial diffusion-weighted (**j**), ADC images (**k**) at the level of pons showing two bilateral symmetric foci of diffusion restriction (high DWI signal and low ADC value) involving reticular formation region (purple arrows). Axial T2W image (**l**) at the same level show two bilateral symmetric foci of high signal intensity in the same corresponding locations in reticular formation (blue arrows).

Three weeks later, trials to wean sedations failed, and the patient had persistent low GCS. A brain CT was done which was unremarkable. Three days later, brain MRI study showed bilateral symmetric cerebral deep white matter confluent hyperintensities in T2W and FLAIR images (Figure 1a–d), with corresponding slightly high signals in DWI. However, no significant low apparent diffusion coefficient (ADC) value was seen (Figure 1e–f). Similar signal abnormalities were seen in the posterior limbs of both internal capsules (Figure 1g–h) and bilateral middle cerebellar peduncles (not shown). MRI features were suggestive of extensive leukoencephalopathy, related to COVID-19 infection. Susceptibility-weighted images (SWI) demonstrated multiple punctate dark blooming foci in the splenium of corpus callosum (Figure 1i), consistent with micro-bleeds attributed to critical illness. Additionally, there were two symmetric spots of diffusion restriction along the rostral pontine reticular formation bilaterally (Figure 1j–k). These spots also showed subtle high T2WI signal intensity (Figure 1l), findings suggestive of bilateral reticular formation (BRF) lesions.

The patient gradually improved and was extubated one week after the brain MRI scan. Two weeks after being extubated and continuously improving, he was transferred to a rehabilitation facility due to generalized muscle weakness and poor balance. One month later, he was discharged from rehabilitation with residual impairment in walking ability.

## Discussion

Following the emergence and global spread of the highly pathogenic coronavirus named SARS‐CoV‐2, different neurological manifestations were encountered in addition to the respiratory involvement in acute pneumonia coronavirus disease 2019 (COVID‐19). Reported neurological manifestations included encephalitis, epileptic seizures, ischaemic/haemorrhagic stroke, delirium, headache and ataxia as well as gustatory and olfactory dysfunction.^1^

While human-to-human transmission of coronaviruses was reported to be by respiratory droplets, the virus also demonstrated tropism for neuronal cells. The mechanisms by which the virus can reach cerebral tissue remain incompletely clear.^1^ However, two hypotheses proposed that coronaviruses may utilize the neuronal-axonal route or the bloodstream to reach the CNS cells.^1,2^

Various neuroimaging features were described in association with COVID-19 infection including MRI findings of different patterns of leukoencephalopathy. The reported patterns of leukoencephalopathy include discrete multifocal white matter hyperintense lesions in FLAIR and DWI with variable enhancement, extensive confluent supratentorial white matter FLAIR hyperintensities, FLAIR hyperintense lesions involving both middle cerebellar peduncles and FLAIR hyperintensities affecting both internal capsules. Patient can have more than one pattern as evident in our case. This leukoencephalopathy was considered non-specific and might represent a hypoxaemia-related late complication in critically ill COVID-19 patients; in a similar fashion to delayed post-hypoxic leukoencephalopathy. However, diffuse leukoencephalopathy in critically ill patients might be seen in direct cerebral infection, sepsis-associated encephalopathy, post-infectious demyelinating or haemorrhagic encephalitis, or toxic encephalopathy.^3,4^ The proinflammatory state in COVID-19 and potentially disrupted blood–brain barrier are speculated to allow the passage of inflammatory and neurotoxic factors into the brain and result in leukoencephalopathy.^3^ Reported intracerebral microhaemorrhages associated with COVID-19 infection can be possibly related to hypoxaemia, disrupted blood–brain barrier or small vessel vasculitis.^4,5^ However, in patients with COVID-19, consumption coagulopathy with increased D-dimer can result in small medullary venous thrombosis with consequent cerebral microbleeds.^6^ High D-dimer was found in our case.

The reticular formation (RF) forms the central core of the brainstem and it contains different interconnected nuclei that help in organizing different functions. At most brainstem levels, the RF can be divided, from medial to lateral, into three longitudinal regions; the raphe nuclei, the medial zone (gigantocellular nuclei) and the lateral zone (parvocellular nuclei). The medial zone represents a source of most of the long ascending and descending projections of the RF.^7^ The lateral zone is mainly related to cranial nerve reflexes and visceral functions. Reticular neurons exhibit extensive and complex axonal projections, reaching various levels in the spinal cord, brainstem, diencephalon and even the cerebral cortex, which contributes to the complexity of the evaluation of this region.^7^

The RF is involved in various functions like taking part in controlling motor responses through reticulospinal tracts, which receive projections from various areas, like basal nuclei, substantia nigra and cerebral cortex.^7^

The RF also participates in modulating information transmission in pain pathways, programming autonomic reflex circuits, and in the control of arousal state and consciousness. Information about different sensory modalities, like pain, are collected into the RF neurons in the midbrain and rostral pons, which are then projected to the thalamic intralaminar nuclei. The latter will project to different regions in the cortex, resulting in heightened arousal after sensory stimuli.^5^ This reticulothalamic pathway in collaboration with monoamine-containing reticular projections are vital for maintaining a normal conscious status. Interestingly, bilateral damage of the brainstem reticular formation neurons and fibers travelling through will end up with prolonged coma. This physiologic input from reticular formation to the cerebrum to sustain a conscious state is named the ascending reticular activating system (ARAS).^5^ Different studies reported an association between consciousness impairment and damaged ARAS parts due to hypoxic ischaemic brain injury.^8,9^ Also, recovery of damaged ARAS with change of related neural connectivity was reported in the recovery of patients with impaired consciousness.^10,11^ Jang et al suggested that thalamocortical projections might be more linked to awareness affection than lower dorsal ARAS.^8^ Injured lower ARAS, connecting the pontine reticular formation and the thalamus, was associated with impaired arousal following hypoxic ischaemic brain injury.^12^

The upper pontine tegmentum role was identified in sustaining consciousness, in comparison with other midbrain structures.^13^ In addition, coma causing lesions were reported to be centred in the upper pontine region, including RF nuclei, and that these lesions can result in coma even without a midbrain damage. Involved nuclei include nucleus pontis oralis that projects to intralaminar nuclei in the thalamus, which in turn projects to cortical regions. Locus coeruleus and raphe complex are other potential pontine nuclei that were also related to maintaining conscious state. Lesions in these nuclei are more likely to result in coma when they are bilateral.^13^ It was suggested that these lesions might either impair the nuclei modulation function on cerebral activity or involve ascending projections from these nuclei or descending fibres from rostral structures that target the upper pons.^13^

We postulate that the BRF hyperintensities encountered in our case probably contributed to the initial refractory impaired consciousness and difficult extubation. This disturbed consciousness might be due to the affection of RF nuclei bilaterally, or ascending or descending projections passing through the region, with consequent impairment on ARAS system. Also, the recovery of the patient might have coincided with healing of these lesions. More neuropathological studies are recommended for radiologic-pathologic correlation and elucidation of the molecular mechanisms of the injuries noted in such patients.

**Figure 2.**
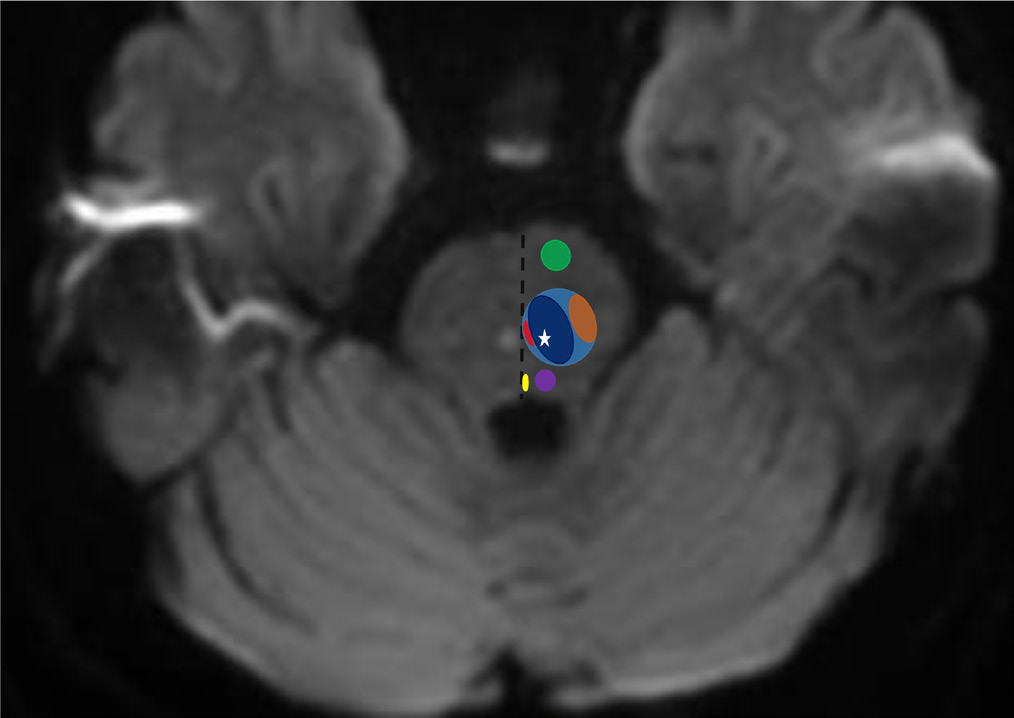
Axial MRI section at the level of pons showing two bilateral symmetric foci of diffusion restriction involving reticular formation region, with a scheme of related pontine regions in the left hemi pons. Descending pyramidal tracts (green oval), medial longitudinal fasciculus (yellow oval), central tegmental tract (purple oval), reticular formation (light blue oval) containing raphe nucleus (red oval), gigantocellular nuclei (dark blue oval) and parvocellular nuclei (orange oval). The hypreintense lesion in the left reticular formation (white star) is likely corresponding to the medial pontine nuclei (gigantocellular nuclei).

Given the proximity of reticular formation to central tegmental tracts (CTT) fibers (Figure 2), which travel through from the red nucleus to reach the inferior olivary nucleus, and connected to the dentate nucleus as a part of Guillain and Mollaret triangle (7), the high pontine T2WI and DWI signal intensity seen in our case could be misdiagnosed to be within the CTT. The CTT lesions reported in the literature were predominantly encountered in pediatric population with epilepsy, hypoxic ischaemic encephalopathy, toxic encephalopathy and different neurodevelopmental disorders.^14^ The specificity and significance of the radiologic findings of CTT lesions and their clinical correlation are not clearly defined. The pathogenesis of CTT lesions in the different clinical conditions was suggested to be related to intramyelination oedema, gliosis or white matter secondary degeneration.^14^

## Learning points

- The reticular formation is a part of the brainstem that is involved in various functions like taking part in the control of arousal state and consciousness.
- Bilateral damage of the reticular formation neurons and fibers travelling through will affect the arousal state and may end up with prolonged coma.
- We postulate that the bilateral affection of the reticular formation, evidenced by bilateral reticular formation hyperintensities in MRI, can be a potential consequence in critically ill COVID-19 patients, especially those with refractory impaired consciousness and difficult extubation.
